# The Physical Treatment of Sciatica

**Published:** 1910-08-27

**Authors:** 


### SURGERY.

### THE PHYSICAL TREATMENT OF SCIATICA.

It is fairly certain that the two things which are most beneficial in a case of sciatica are rest and time; all sorts of drugs have been employed, but none can be called specific. It is important in most cases, however, to be able to do something locally, and, whatever internal remedies may be used, physical methods are likely to appeal to the patient.

Most of the physical methods act by producing some change in the circulation, either by variation in temperature or by means of the electric current, or by chemical or mechanical means. Broadly speaking, one may say that the more acute the case the less active should the therapeutic measures be. In the most acute cases of sciatica rest in bed is essential, and the external applications that are most likely to be of benefit are for the most part local applications of either heat or cold. Hot poultices are an old and useful remedy, and so also is hot ironing over brown paper that has been steeped in vinegar and water. Hot lead and opium fomentations are also good, and they seem to act in consequence of the small amount of opium they contain rather more favourably than do hot fomentations containing no opium. Some observers apply cold by means of ice poultices or by a spray of methyl chloride along the course of the nerve, and some like to use alternate applications of cold and of heat, the essential thing being to vary the circulation in the part. It is on account of their influence on the circulation that the more modern forms of heat application by means of radiant electric heat baths, fango-packs, and hot sand baths are sometimes used instead of the older fashioned but equally beneficial methods. Plasters and acupuncture act in much the same way also, but their effects are transient, and therefore, although they may be resorted to at some stage or another in the treatment of sciatica, it is unlikely that they will be persisted with, because it is not easy to continue with the plasters or acupuncture very often. Dry-cupping is a process, on the other hand, which can be readily repeated, and it acts almost as well as plastering. It is surprising that it is not more often employed in the relief of the pain in acute sciatica. Another method of relieving the acute pain is to apply cold-water bandages to the part, or bandages previously soaked in cold water containing a little alcohol and covered over with oilskin or gutta-percha tissue; these water-packs rapidly become warm, and the relief to the pain is often so marked that by their use the patient may get a good night's rest. Some authorities apply massage in the acute cases, but it is generally to be avoided at this stage, especially as the hard pressure that is employed by masseurs may considerably aggravate the pain. Very light friction carefully applied by the doctor himself is sometimes beneficial, especially if the most painful spots are avoided.

As regards electrical treatment, it is not to be recommended in the acute stage except with considerable caution. If it is employed at all it should take the form of the galvanic current. Two equal-sized electrodes, each ten inches square, should be employed, the negative being placed on the abdomen and the positive on the posterior iliac spine or the sciatic notch; both may be moved evenly along the leg without interruption of the current. It is often advisable to use only 3 milliampères of current or less, and never more than 8 to 10 milliampères. Some authorities recommend high-frequency currents in these cases, but opinions are very much divided as to the benefits to be derived from them in acute sciatica.

While the application of physical methods of treatment in the acute cases of sciatica is limited, it has ample scope when the malady has become subacute or chronic. Massage is probably the commonest form of treatment. Very light rubbing should be used to begin with, and as improvement takes place the massage may be more energetic. Only when the way has been carefully felt should tapotement, intermittent pressure, and vibrations over the painful spots be resorted to. It is sometimes advisable to extend the séance for longer than fifteen minutes daily; too long an application makes the patients irritable. When the pain has considerably lessened, modified gymnastic exercises sometimes do much good, though here again it is most important not to overdo them. The patient should stand at such a distance from the door that he can easily touch the key-hole with his sound foot. He then lifts the affected leg as high as possible without bending the knee, pressing the sole of the foot against the door. The height obtained can be marked with a pencil, and the patient must try to get a little higher every day. A similar result may be obtained passively by lifting the stretched leg as high as possible while the patient is lying quietly on his back, but the active stretching is to be preferred owing to the fact that the passive form, if not given very cautiously, may go too far and throw the condition back, whereas the patient himself will not be nearly so liable to overdo the practice of active exercises.

Hydrotherapy may well be combined with the massage and exercises, though some authorities prefer to give the massage and the douches separately. In the wet massage of Vichy and Aix-les-Bains it not infrequently happens that the hands of the masseur slip, so that it is difficult to obtain an exact and deep effect; on this account it is sometimes advisable to order the douche baths separately, either immediately before or after the massage. There are several different methods of hydrotherapy that might be employed. Plain reclining baths often suit patients very well; they should be 94° F. at the beginning, and of fifteen minutes duration to start with; during the course of treatment both temperature and duration may be increased—temperature to 99° F. or 100° F., and the duration to half an hour—after that one hour's rest is absolutely necessary. Chemical substances such as brine, pine extract, and ammonia may be added to the water of the bath, or, as in the Nauheim baths, which should be given for a shorter time than the others, miliary massage by means of carbon dioxide bubbles, producing active hyperæmia of the skin, may prove valuable. Hot sitz baths are contra-indicated, owing to the overflexing of the thigh required in the position adopted in these baths. Turkish and electric light baths act chiefly by producing local extreme perspiration with consequent active hyperæmia of the vessels from which the perspiration comes, and there seems to be no specific reason for their employment rather than other forms of local heat.

It may be urged that many of the methods of physical treatment of sciatica necessitate expense; this, however, is not essential; and although it may be better for the patient when possible to have special apparatus for the treatment or to go to places where such special apparatus is used, it is possible to improvise much at home, even in poor districts.

Hot mashed potatoes put into a woollen cloth are an excellent material for hot poultices, and are often employed in poor districts. Packs to be applied to the hip or leg require only some yards of old linen ten to fifteen inches wide, and a suitable piece of flannel as a cover can be procured very easily. As regards the douches, if there is a bathroom in the patient's house the doctor may easily attach an indiarubber tube to the tap and employ almost any kind of douche. Even when no bathroom is available a big watering-pot, the rose of which has been removed, enables one to give a douche of any temperature, and by taking two, the one filled with hot and the other with cold water, one may improvise a very good substitute for the Scotch douche.

